# Cognitive Intervention As an Early Non-pharmacological Strategy in Alzheimer's Disease: A Translational Perspective

**DOI:** 10.3389/fnagi.2016.00280

**Published:** 2016-11-29

**Authors:** Sarah W. Gehres, Andreia Rocha, Antoine Leuzy, Cássio M. Loss, Giordano G. Viola, Eduardo R. Zimmer

**Affiliations:** ^1^Department of Biochemistry, Federal University of Rio Grande do SulPorto Alegre, Brazil; ^2^Department NVS, Center for Alzheimer Research, Division of Translational Alzheimer Neurobiology, Karolinska InstitutetStockholm, Sweden; ^3^Department of Physiology, Federal University of SergipeSão Cristóvão, Brazil; ^4^Brain Institute of Rio Grande do Sul (BraIns), Pontifical Catholic University of Rio Grande do SulPorto Alegre, Brazil

**Keywords:** Alzheimer's disease, cognitive intervention, cognitive reserve, dementia, environmental enrichment

Brain amyloid-β (Aβ) accumulation is currently considered the main causative pathophysiological event in Alzheimer's disease (AD) (Hardy and Higgins, 1992; Karran et al., 2011). Importantly, this process is thought to precede the onset of AD clinical symptoms by more than two decades, indicating that early therapeutic strategies prior to symptomatology offer the best chance of success. In line with this, there is growing attention being paid to the concept of cognitive reserve (CR) (Stern et al., 1994; Stern, 2002). CR concept is based on extensive epidemiological data indicating that those with higher lifetime levels of social, physical, and cognitive engagement have a lower risk of developing dementia despite the presence of brain pathology (Fratiglioni et al., 2004; Nithianantharajah and Hannan, 2009). Recently, cognitive intervention (CI)—such as cognitive training (Bahar-Fuchs et al., 2013), cognitive stimulation (Woods et al., 2012), and cognitive rehabilitation (Clare et al., 2003)—has emerged as a potential non-pharmacological strategy for the treatment and prevention of AD (Gates and Sachdev, 2014). Although based upon distinct theoretical constructs, these CI strategies are frequently not distinguished in clinical trials.

Clinical data are supported by compelling evidence from experimental studies, which have demonstrated that early-life exposure to environmental enrichment (EE), an experimental method of CR in animals, is able to prevent memory decline in AD-like animal models (Valero et al., 2011; Verret et al., 2013; Polito et al., 2014). However, the precise mechanisms behind this phenomenon remain elusive. Environmental enrichment for animals involves stimulating not only their senses (e.g., smell, sight, touch), but also stimulating their ability to learn and adapt when exposed to novelty and challenge, including via stimulation of innate behaviors, such as foraging and partner seeking. The same line of thinking can be applied to humans, on its own scale. This would translate into exercise, social interaction, learning new things, exploring new environments, keeping the brain active with cognitive training, i.e., challenging the brain to self-adapt to novelty.

In a recent study, published in *Frontiers in Aging Neuroscience*, Bezzina et al. (2015) reared mice harboring a human pathological double mutation in the amyloid precursor protein gene (APP, model tg2576) in standard or enriched housing conditions for 10 weeks, starting at 3 months of age (pre-Aβ plaque phase). Two weeks after EE exposure, transgenic animals housed in standard or EE cages presented similar seizure susceptibility to pentylenetetrazole (PTZ), a well-established GABA receptor antagonist. Moreover, the frequency of interictal spikes—indexed by electroencephalography (EEG)—after EE was similar between groups. In short, the authors show that EE is not capable of halting the Aβ-induced aberrant neuronal activity in the Tg2576 AD-like model. These findings indicate that preventive effects of EE on cognitive decline are not necessarily related to changes in neuronal activity. Although negative, these findings are very relevant since they help rule out abnormal neuronal activity as a mechanism by which EE exerts its beneficial impact on memory performance.

By contrast, data analyzing the effects of EE on neurogenesis in AD-like animal models have been mostly positive. EE exposure for 6 months was able to restore the impaired hippocampal neurogenesis of a triple transgenic AD-like mouse model (3xTg-AD, a model harboring human mutations in: APP (Swedish, KM670/671NL), Tau (MAPT P301L) and Presenilin 1 (PSEN1 M146V) (Rodríguez et al., 2008, 2011). The density analysis of hippocampal proliferating cells was performed by immunohistochemistry targeting the presence of phosphorylated Histone H3 (HH3), and their potential neuronal and glial phenotype by co-localizing the proliferating cells with the immature neuronal marker doublecortin (DCX), the mature neuronal marker (NeuN) or the specific astroglial marker (GFAP). This very interesting study showed a significant increase in neuronal proliferating cells (HH3/DCX or HH3/NeuN positive) in the dentate gyrus of transgenic animals housed in EE. These results, together with the findings of other experimental studies, suggest the stimulation of adult neurogenesis as a potential process by which EE prevents cognitive impairment in AD-like models (Wolf et al., 2006; Herring et al., 2009; Mirochnic et al., 2009; Lahiani-Cohen et al., 2011). In this context, recent evidence indicates that the benefitial effects of EE are more related to the reorganization of the neuronal network built by these newborn neurons than to the absolute number of newborn neurons *per se* (Vivar et al., 2016).

However, the impact of EE on Aβ levels in the brain is still largely inconclusive (Mainardi et al., 2014; Polito et al., 2014; Rodríguez et al., 2015). Male APP23, a mouse model expressing the human APP Swedish double mutation, exposed to EE starting at 3 months of age until 7 months, or 18 months, showed a marked cognitive improvement without altering soluble Aβ (7 month-old) but reducing the number of Aβ plaques at 18 month-old. These data indicate that EE effect seems to affect Aβ in a distinct manner.

We are of the opinion that deciphering the underlying mechanisms of EE can provide important information about novel therapeutic targets. With currently available treatments for AD being palliative at best and ineffective at worst—results from drug trials targeting brain Aβ have been either inconclusive or negative—non-pharmacological interventions stimulating cognition in humans hold a measure of promise, not only for delaying symptomatic onset and the loss of cognitive functions, but also in terms of unveiling potential targets.

The take-home message from EE studies is clear, however, and translates to human disease: interventions must occur early on in the disease course, prior to substantial cognitive deficits (Figure 1). The question then becomes: how to translate these findings to clinical research? A promising approach is to study individuals in the so-called “preclinical” or “asymptomatic at-risk” stage of AD [please see revised definition of preclinical AD in Sperling et al. (2011) and Dubois et al. (2016)]. Defined by the absence of cognitive deficits despite *in situ* AD pathology—established using cerebrospinal fluid (CSF) or positron emission tomography (PET) based biomarkers—cross-sectional and longitudinal studies suggest this preclinical phase to span some 20–30 years (Jansen et al., 2015). Based on this, non-pharmacological interventions directed at CR could be encouraged in so called “amyloid-positive” individuals believed to be on the path to symptomatic AD. In this way, we refer to the concepts of primary and secondary prevention, as defined by Gates et al. (2011). Primary prevention in the form of early intervention, aimed at preclinical individuals, in order to keep them asymptomatic for as long as possible; and secondary prevention in the form of cognitive training and stimulation for those who already show subtle and early cognitive impairment [such as those in the mild cognitive impairment (MCI) phase], to slow the cognitive decline into AD dementia.

**Figure 1:**
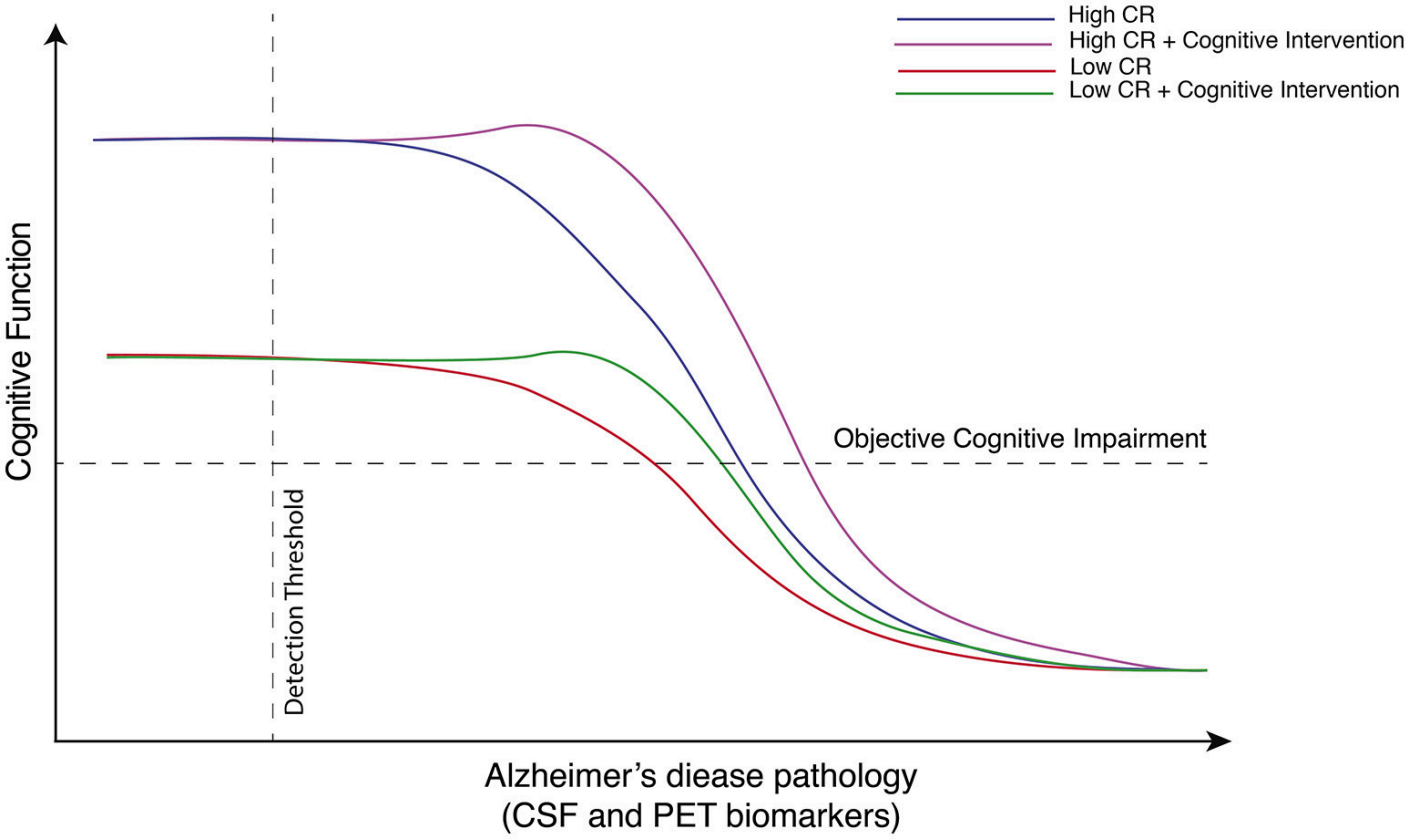
**Hypothetical model depicting the beneficial effects of cognitive intervention on cognitive function on low and high cognitive reserve individuals as a function of Alzheimer's disease neuropathology**. This hypothetical model exemplifies changes in cognitive function changes over Alzheimer's disease (AD) continuum in individuals with high and low cognitive reserve (CR). When AD pathology is absent or possibly below detectable levels using current biomarkers, individuals with high CR have been shown to perform better on neurophysiological tests than individual with low CR. While cognitive intervention probably cannot ultimately arrest the progression of AD pathology, as measured by imaging or fluid based biomarkers, it can potentially delay the onset and progression of cognitive symptoms. In keeping with the observation that individuals with high CR can tolerate greater levels of pathology prior to declines in cognitive performance, the rate of decline is greater in the high CR group.

While follow-up studies with longer assessment periods are essential for more definitive data, early results from several multi-domain non-pharmacologic intervention studies—addressing diet, exercise, cognitive training and vascular risk monitoring—suggest that cognitive functioning can be maintained or even improved in at-risk elderly individuals (Ngandu et al., 2015). The incorporation of amyloid biomarkers into such intervention trials could stand as a valuable adjunct to cognitive and functional outcome measures, possibly allowing for more rapid translational estimations.

Though EE stimulation has been shown to be a promising approach for delaying disease progression in animal models mimicking early onset autosomal dominant AD, which accounts for less than 5% of cases, what about the far more common age-related (sporadic) and multifactorial form of AD? How does EE impact on sporadic models of AD? Here stands a problem in that models replicating sporadic AD are currently lacking. In our opinion, major developments in modeling the sporadic form of AD in rodents will potentially come from the incorporation of genetic variations that increase the risk of developing AD, such as the ε4 allele of apolipoprotein E (APOE) gene, or the R47H allele of the triggering receptor expressed on myeloid cells 2 (TREM2). Animal models expressing such combinations of genetic risk factors may better resemble sporadic AD and ultimately translate into more relevant disease models.

In conclusion, study designs aiming toward deciphering EE specific mechanisms are vital and can potentially drive non-pharmacological strategies. Here we make an argument that the combination of EE with models that better mimic the sporadic form of AD, as well as CSF- and PET-based biomarkers, will potentially provide important insights into AD pathophysiology and highlight novel therapeutic targets, with strong translational value.

## Author contributions

SG and AR were responsible for reviewing the literature and drafting the manuscript. CL, GV and AL were responsible for revising the manuscript. EZ was responsible for the conceptualization and drafting the manuscript. All authors critically revised the final version of the manuscript.

### Conflict of interest statement

The authors declare that the research was conducted in the absence of any commercial or financial relationships that could be construed as a potential conflict of interest.
